# Ethyl 1*H*-indole-2-carboxyl­ate

**DOI:** 10.1107/S2414314620012055

**Published:** 2020-09-08

**Authors:** Will E. Lynch, Christine R. Whitlock, Clifford W. Padgett

**Affiliations:** a Georgia Southern University, Department of Chemistry and Biochemistry, Box 8064, Statesboro, GA 30460, USA; Benemérita Universidad Autónoma de Puebla, México

**Keywords:** crystal structure, indole, hydrogen bonding

## Abstract

Our work in the area of synthesis of tris indole compounds as a potential chelator led to the synthesis and crystallization of ethyl 1*H*-indole-2-carboxyl­ate, C_11_H_11_NO_2_, an indole that was synthesized by the thionyl chloride reaction of 1*H*-indole-2-carb­oxy­lic acid, followed by dissolution in ethanol. The mol­ecular packing exhibits a herringbone pattern with the zigzag running along the *b*-axis direction; the compound crystallizes as a hydrogen-bonded dimer resulting from O⋯H—N hydrogen bonds, between the indole N—H group and the keto oxygen atom, which build centrosymmetric *R* ^2^_2_(10) ring motifs in the crystal.

## Abstract 2

The synthesis and structure of 1*H*-indole-2-carboxyl­ate is presented.

## Graphical abstract

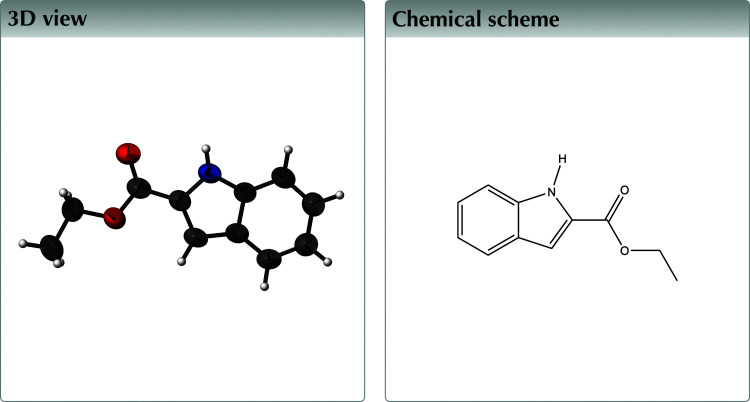

## Structure description

Indole esters can easily be prepared from 1*H-*indole-2-carb­oxy­lic acid *via* an isolated acyl chloride inter­mediate followed by dissolving the residue in the appropriate alcohol solvent. These indole-type compounds are of inter­est because of their prevalence in nature (Stempel & Gaich, 2016 ▸). Derivatives of this type of compound have also been implicated in a number of biological roles including anti­fungal (Kipp *et al.*, 1999 ▸), anti­tumor (Lu *et al.*, 2016 ▸) and anti-inflammatory (Liu *et al.*, 2016 ▸) agents. These types of compounds have also been reported as potential cellular inhibitors of kinase (Jobson *et al.*, 2009 ▸) as well as an antagonist for glycine-binding sites (Ohtani *et al.*, 2002 ▸). Previous reports include the structures of indole-2-carb­oxy­lic acid (Morzyk-Ociepa *et al.*, 2004 ▸) and methyl 1*H*-indole-2-carboxyl­ate (Almutairi *et al.*, 2017 ▸).

**Figure 1:**
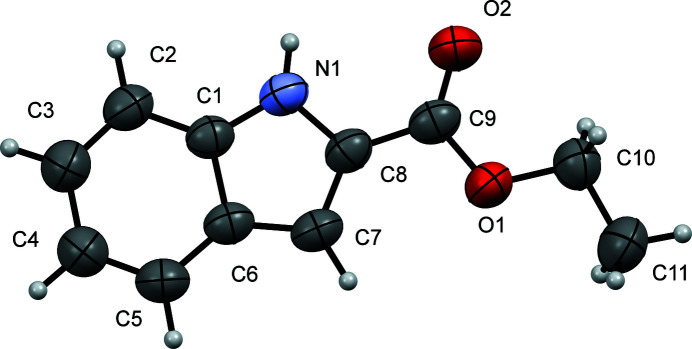
A view of the mol­ecular structure of the title compound, with the atom labelling. Displacement ellipsoids are drawn at the 50% probability level.

**Figure 2:**
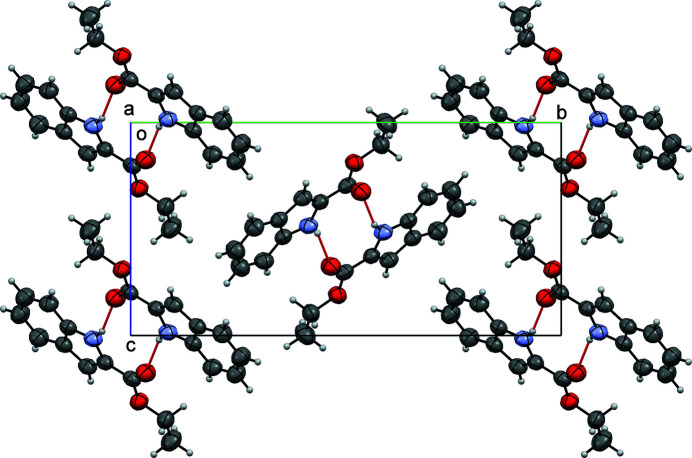
Crystal packing diagram of title compound viewed along [100]. Hydrogen bonds are coloured red.

Herein we report the crystal structure of ethyl 1*H*-indole-2-carboxyl­ate (Fig. 1 ▸), which forms a hydrogen-bonded dimer. The hydrogen bonding occurs between N atoms of the indole ring and the keto oxygen atoms with an *R*(10) synthon. The hydrogen bond between N1 and O2^i^ is characterized by an N⋯O separation of 2.877 (3) Å [symmetry code: (i) −*x* + 2, −*y* + 1, −*z* + 1; Table 1 ▸], and the ring motifs, 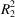 (10), are placed on inversion centres in the space group *P*2_1_/*c* (Fig. 2 ▸). The crystal structure exhibits a classic herringbone pattern (Fig. 2 ▸) with the blocks consisting of the hydrogen-bonded dimers, with the zigzag running along the *b*-axis direction. The mol­ecule is nearly planar, with a r.m.s.d. of 0.028 Å for the non-hydrogen atoms. There are no other short contacts or π–π inter­actions observed in the crystal.

## Synthesis and crystallization

The title compound was synthesized by modification of an early method laid out by Terent’ev *et al.* (1969 ▸). Indole-2-carb­oxy­lic acid (0.50 g, 3.1 mmol) was dissolved in SOCl_2_ (19 ml) at 0°C. After stirring for 1 h, the solution was rotary evaporated and to the resulting oil was added absolute ethanol (17 ml) at room temperature. After stirring overnight, the solution was vacuum filtered to yield ethyl 1*H*-indole-2-carboxyl­ate as a beige solid, which was recrystallized from methanol to yield 0.54 g (2.9 mmol, 93%) of the product. Further recrystallization by slow evaporation from methanol solution resulted in X-ray quality crystals.

## Refinement

Crystal data, data collection and structure refinement details are summarized in Table 2 ▸.

## Supplementary Material

Crystal structure: contains datablock(s) I. DOI: 10.1107/S2414314620012055/bh4054sup1.cif

Structure factors: contains datablock(s) I. DOI: 10.1107/S2414314620012055/bh4054Isup2.hkl

Supporting information file. DOI: 10.1107/S2414314620012055/bh4054Isup3.cml

CCDC reference: 2026531

Additional supporting information:  crystallographic information; 3D view; checkCIF report

## Figures and Tables

**Table 1:** Hydrogen-bond geometry (Å, °)

*D*—H⋯*A*	*D*—H	H⋯*A*	*D*⋯*A*	*D*—H⋯*A*
N1—H1⋯O2^i^	0.84 (3)	2.08 (3)	2.877 (3)	158 (3)

Symmetry code: (i) 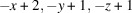 .

**Table 2:** Experimental details

Crystal data	
Chemical formula	C_11_H_11_NO_2_
*M*_r_	189.21
Crystal system, space group	Monoclinic, *P*2_1_/*c*
Temperature (K)	170
*a*, *b*, *c* (Å)	5.5622 (7), 18.891 (2), 9.6524 (13)
β (°)	104.454 (13)
*V* (Å^3^)	982.1 (2)
*Z*	4
Radiation type	Mo *K*α
μ (mm^−1^)	0.09
Crystal size (mm)	0.4 × 0.05 × 0.05

Data collection	
Diffractometer	Rigaku XtaLAB mini
Absorption correction	Multi-scan (*CrysAlis PRO*; Rigaku OD, 2018 ▸)
*T*_min_, *T*_max_	0.998, 1.000
No. of measured, independent and observed [*I* > 2σ(*I*)] reflections	5586, 1804, 991
*R*_int_	0.047
(sin θ/λ)_max_ (Å^−1^)	0.602

Refinement	
*R*[*F* ^2^ > 2σ(*F* ^2^)], *wR*(*F* ^2^), *S*	0.049, 0.144, 1.01
No. of reflections	1804
No. of parameters	132
H-atom treatment	H atoms treated by a mixture of independent and constrained refinement
Δρ_max_, Δρ_min_ (e Å^−3^)	0.28, −0.16

Computer programs: *CrysAlis PRO* (Rigaku OD, 2018 ▸), *SHELXT* (Sheldrick, 2015*a* ▸), *SHELXL* (Sheldrick, 2015*b* ▸) and *OLEX2* (Dolomanov *et al.*, 2009 ▸).

## full crystallographic data

### Crystal data

**Table:**

C_11_H_11_NO_2_	*F*(000) = 400
*M**_r_* = 189.21	*D*_x_ = 1.280 Mg m^−^^3^
Monoclinic, *P*2_1_/*c*	Mo *K*α radiation, λ = 0.71073 Å
*a* = 5.5622 (7) Å	Cell parameters from 611 reflections
*b* = 18.891 (2) Å	θ = 2.4–21.1°
*c* = 9.6524 (13) Å	µ = 0.09 mm^−^^1^
β = 104.454 (13)°	*T* = 170 K
*V* = 982.1 (2) Å^3^	Needle, colourless
*Z* = 4	0.4 × 0.05 × 0.05 mm

### Data collection

**Table:**

Rigaku XtaLAB mini diffractometer	1804 independent reflections
Radiation source: fine-focus sealed X-ray tube, Rigaku (Mo) X-ray Source	991 reflections with *I* > 2σ(*I*)
Graphite monochromator	*R*_int_ = 0.047
ω scans	θ_max_ = 25.3°, θ_min_ = 2.2°
Absorption correction: multi-scan (CrysAlisPro; Rigaku OD, 2018)	*h* = −6→6
*T*_min_ = 0.998, *T*_max_ = 1.000	*k* = −22→22
5586 measured reflections	*l* = −6→11

### Refinement

**Table:**

Refinement on *F*^2^	Primary atom site location: dual
Least-squares matrix: full	Secondary atom site location: difference Fourier map
*R*[*F*^2^ > 2σ(*F*^2^)] = 0.049	Hydrogen site location: mixed
*wR*(*F*^2^) = 0.144	H atoms treated by a mixture of independent and constrained refinement
*S* = 1.01	*w* = 1/[σ^2^(*F*_o_^2^) + (0.0611*P*)^2^] where *P* = (*F*_o_^2^ + 2*F*_c_^2^)/3
1804 reflections	(Δ/σ)_max_ < 0.001
132 parameters	Δρ_max_ = 0.28 e Å^−^^3^
0 restraints	Δρ_min_ = −0.16 e Å^−^^3^

### Special details

**Table:**

Refinement. All carbon-bound H atoms were positioned geometrically and refined as riding, with C—H = 0.95, 0.98 or 0.99 Å and *U*_iso_(H) = 1.2*U*_eq_(C) or *U*_iso_(H) = 1.5*U*_eq_(C) for C(H) and CH_3_ groups, respectively. Hydrogen atom of the N—H group was refined freely.

### Fractional atomic coordinates and isotropic or equivalent isotropic displacement parameters (Å^2^)

**Table:**

	*x*	*y*	*z*	*U*_iso_*/*U*_eq_	
O1	0.4202 (3)	0.51968 (8)	0.18977 (19)	0.0674 (5)	
O2	0.8074 (3)	0.53509 (10)	0.32447 (19)	0.0769 (6)	
N1	0.7445 (4)	0.41313 (12)	0.4844 (2)	0.0618 (6)	
C1	0.6528 (4)	0.35476 (13)	0.5376 (2)	0.0546 (6)	
C6	0.4072 (4)	0.34466 (13)	0.4568 (2)	0.0561 (6)	
C9	0.6121 (5)	0.50269 (13)	0.2963 (3)	0.0603 (7)	
C8	0.5633 (4)	0.44041 (13)	0.3735 (3)	0.0559 (6)	
C7	0.3542 (4)	0.39987 (13)	0.3545 (3)	0.0604 (7)	
H7	0.201542	0.407332	0.285492	0.072*	
C2	0.7649 (5)	0.30983 (14)	0.6497 (3)	0.0667 (7)	
H2	0.931108	0.317126	0.703333	0.080*	
C10	0.4457 (5)	0.58135 (13)	0.1043 (3)	0.0713 (8)	
H10A	0.570546	0.572318	0.049089	0.086*	
H10B	0.499351	0.622958	0.166698	0.086*	
C5	0.2734 (5)	0.28695 (15)	0.4909 (3)	0.0713 (8)	
H5	0.107754	0.278384	0.437693	0.086*	
C3	0.6258 (5)	0.25482 (14)	0.6791 (3)	0.0745 (8)	
H3	0.696946	0.223507	0.755363	0.089*	
C4	0.3833 (5)	0.24349 (15)	0.6006 (3)	0.0760 (8)	
H4	0.292584	0.204613	0.624028	0.091*	
C11	0.1971 (5)	0.59444 (16)	0.0056 (3)	0.0935 (10)	
H11A	0.149807	0.553780	−0.058508	0.140*	
H11B	0.204101	0.637068	−0.051138	0.140*	
H11C	0.073960	0.601063	0.061479	0.140*	
H1	0.879 (5)	0.4339 (15)	0.520 (3)	0.087 (10)*	

### Atomic displacement parameters (Å^2^)

**Table:**

	*U*^11^	*U*^22^	*U*^33^	*U*^12^	*U*^13^	*U*^23^
O1	0.0631 (11)	0.0688 (12)	0.0625 (11)	−0.0022 (9)	0.0009 (9)	0.0064 (9)
O2	0.0672 (13)	0.0828 (13)	0.0715 (13)	−0.0150 (10)	0.0001 (10)	0.0024 (10)
N1	0.0537 (14)	0.0712 (15)	0.0544 (13)	−0.0052 (12)	0.0021 (12)	−0.0023 (12)
C1	0.0522 (15)	0.0606 (16)	0.0496 (14)	−0.0010 (12)	0.0101 (12)	−0.0062 (13)
C6	0.0500 (15)	0.0628 (15)	0.0530 (14)	0.0000 (12)	0.0082 (12)	−0.0084 (13)
C9	0.0573 (17)	0.0670 (17)	0.0524 (15)	0.0007 (14)	0.0054 (14)	−0.0125 (14)
C8	0.0564 (16)	0.0586 (15)	0.0486 (14)	0.0021 (12)	0.0054 (12)	−0.0056 (13)
C7	0.0487 (15)	0.0709 (17)	0.0557 (15)	0.0014 (13)	0.0021 (12)	−0.0063 (14)
C2	0.0571 (16)	0.0769 (18)	0.0607 (17)	0.0033 (14)	0.0046 (13)	0.0009 (15)
C10	0.0766 (19)	0.0626 (17)	0.0715 (18)	−0.0028 (14)	0.0127 (15)	0.0068 (14)
C5	0.0549 (16)	0.0793 (18)	0.0742 (19)	−0.0090 (14)	0.0056 (14)	−0.0001 (16)
C3	0.0708 (19)	0.0766 (19)	0.0727 (19)	0.0011 (15)	0.0115 (16)	0.0111 (15)
C4	0.0697 (19)	0.0777 (19)	0.078 (2)	−0.0072 (14)	0.0136 (16)	0.0097 (16)
C11	0.088 (2)	0.089 (2)	0.091 (2)	0.0041 (17)	−0.0015 (18)	0.0239 (18)

### Geometric parameters (Å, º)

**Table:**

O1—C9	1.324 (3)	C2—H2	0.9500
O1—C10	1.455 (3)	C2—C3	1.367 (3)
O2—C9	1.217 (3)	C10—H10A	0.9900
N1—C1	1.368 (3)	C10—H10B	0.9900
N1—C8	1.374 (3)	C10—C11	1.491 (3)
N1—H1	0.84 (3)	C5—H5	0.9500
C1—C6	1.406 (3)	C5—C4	1.359 (4)
C1—C2	1.394 (3)	C3—H3	0.9500
C6—C7	1.416 (3)	C3—C4	1.389 (4)
C6—C5	1.404 (3)	C4—H4	0.9500
C9—C8	1.454 (3)	C11—H11A	0.9800
C8—C7	1.366 (3)	C11—H11B	0.9800
C7—H7	0.9500	C11—H11C	0.9800
			
C9—O1—C10	117.45 (19)	O1—C10—H10A	110.4
C1—N1—C8	108.9 (2)	O1—C10—H10B	110.4
C1—N1—H1	127 (2)	O1—C10—C11	106.8 (2)
C8—N1—H1	123 (2)	H10A—C10—H10B	108.6
N1—C1—C6	107.6 (2)	C11—C10—H10A	110.4
N1—C1—C2	130.2 (2)	C11—C10—H10B	110.4
C2—C1—C6	122.2 (2)	C6—C5—H5	120.3
C1—C6—C7	106.9 (2)	C4—C5—C6	119.3 (2)
C5—C6—C1	118.3 (2)	C4—C5—H5	120.3
C5—C6—C7	134.8 (2)	C2—C3—H3	119.1
O1—C9—C8	112.1 (2)	C2—C3—C4	121.8 (3)
O2—C9—O1	123.6 (2)	C4—C3—H3	119.1
O2—C9—C8	124.3 (2)	C5—C4—C3	121.3 (3)
N1—C8—C9	120.5 (2)	C5—C4—H4	119.4
C7—C8—N1	109.2 (2)	C3—C4—H4	119.4
C7—C8—C9	130.3 (2)	C10—C11—H11A	109.5
C6—C7—H7	126.4	C10—C11—H11B	109.5
C8—C7—C6	107.3 (2)	C10—C11—H11C	109.5
C8—C7—H7	126.4	H11A—C11—H11B	109.5
C1—C2—H2	121.4	H11A—C11—H11C	109.5
C3—C2—C1	117.2 (2)	H11B—C11—H11C	109.5
C3—C2—H2	121.4		

### Hydrogen-bond geometry (Å, º)

**Table:**

*D*—H···*A*	*D*—H	H···*A*	*D*···*A*	*D*—H···*A*
N1—H1···O2^i^	0.84 (3)	2.08 (3)	2.877 (3)	158 (3)

Symmetry code: (i) −*x*+2, −*y*+1, −*z*+1.
